# Extrarenal papillary renal cell carcinoma without a renal primary: a case report

**DOI:** 10.1097/RC9.0000000000000638

**Published:** 2026-07-28

**Authors:** Analaura Del Rivero-Morfin, Carlos A. Gonzalez-Assad, Eduardo A. Guzman-Huerta, Jesus A. Limon-Rodriguez

**Affiliations:** a Surgical Oncology Research Committee, Department of Surgical Oncology, Tecnologico de Monterrey, School of Medicine and Health Sciences, Monterrey, N.L., Mexico; b Department of Surgical Oncology, Tecnologico de Monterrey, School of Medicine and Health Sciences. Monterrey, N.L., Mexico; c Department of Medical Oncology, AUNA Oncology Center Monterrey. Monterrey, N.L., Mexico

**Keywords:** extrarenal, immunotherapy, nivolumab, non-clear cell RCC, papillary renal cell carcinoma, surgical oncology

## Abstract

**Introduction and importance:**

Primary extrarenal papillary renal cell carcinoma (pRCC) without an identifiable renal lesion is exceedingly rare, with fewer than ten cases reported in the literature. From a surgical standpoint, it poses a dual challenge: establishing an accurate diagnosis and determining the extent of resection in the absence of a renal primary lesion.

**Case presentation:**

We report the case of a 77-year-old male who was diagnosed with a solitary retroperitoneal extrarenal mass, confirmed by histopathology as a type 2 pRCC. He underwent laparoscopic resection of the mass; however, early postoperative follow-up demonstrated disease progression with pulmonary lesions. Systemic immunotherapy was initiated with nivolumab, achieving a partial response at the 3-month follow-up and stable disease at 12 months.

**Clinical discussion:**

Given the limited number of reported cases, the optimal management of extrarenal pRCC remains unclear. This case supports the potential role of immunotherapy and highlights the diagnostic and therapeutic challenges of such rare presentations.

**Conclusion:**

Extrarenal pRCC is a rare and challenging diagnostic entity. Multidisciplinary evaluation – including contrast-enhanced imaging, pathology, and surgical and medical oncology – is essential for accurate management. Immunotherapy may represent a therapeutic option in selected cases.

## Introduction

Papillary renal cell carcinoma (pRCC) is an aggressive subtype of renal cell carcinoma, associated with a worse prognosis than other histological subtypes[1]. Among the two subclassifications of pRCC, type 2 is associated with a higher nuclear grade, a more advanced stage at presentation, and worse outcomes compared with type 1[2].

Although pRCC classically arises from the renal cortex, rare instances of extrarenal pRCC, defined as tumors without an identifiable renal primary, have been described[3]. These cases are exceedingly rare and lack standardized management guidelines[4].

HIGHLIGHTS

- Extrarenal PRCC without an identifiable renal primary is extremely rare.
- Laparoscopic resection is feasible for selected retroperitoneal masses, providing both a definitive diagnosis and local disease control.
- Immune checkpoint inhibitors, such as nivolumab, may provide disease control in metastatic non-clear cell RCC.
- Multidisciplinary management is essential in rare presentations of RCC.

We present the case of a 77-year-old man with a solitary type 2 retroperitoneal pRCC, without an identifiable renal primary, who underwent laparoscopic resection followed by nivolumab immunotherapy with a favorable clinical response[5]. This case report has been documented in line with the SCARE checklist[6].

## Case presentation

A 77-year-old man with a past medical history of systemic hypertension, treated with antihypertensive medication, presented to the emergency department with bilateral lower-extremity edema. No associated symptoms were reported, and the physical examination was unremarkable. He denied tobacco, alcohol, or recreational drug use and reported no known drug allergies. The patient was admitted for further diagnostic evaluation.

**Figure 1.**
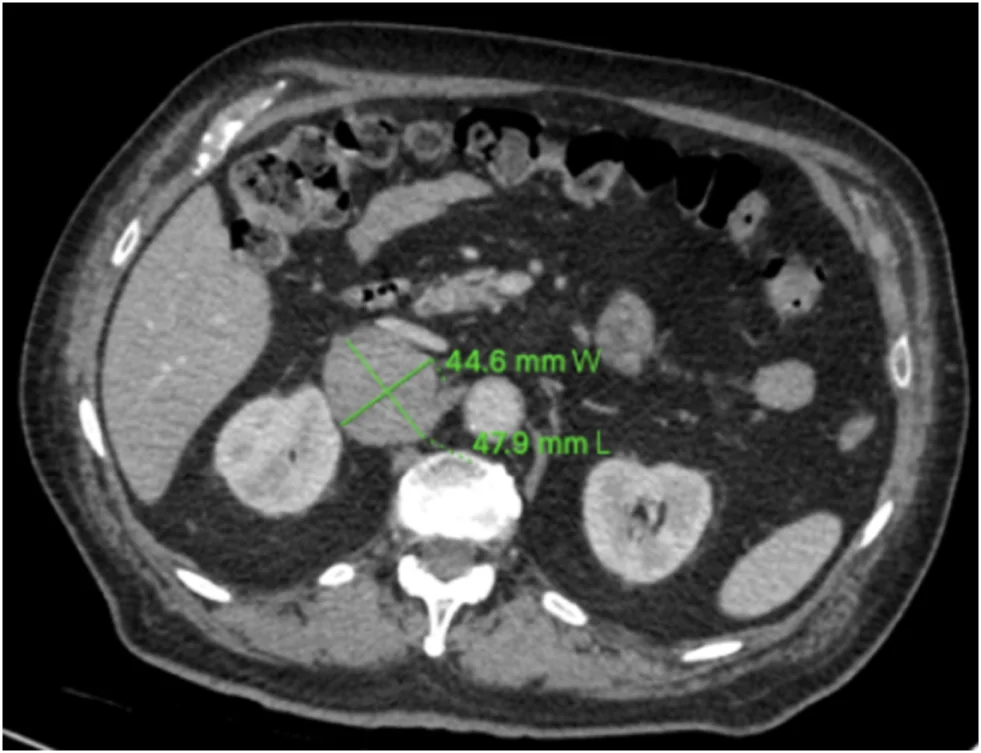
First CT scan.

Initial laboratory studies were within normal limits. An abdominopelvic contrast-enhanced computed tomography (CT) scan revealed a single, well-defined retroperitoneal solid lesion measuring 48 × 45 mm, adjacent to the right kidney and adrenal gland (Fig. 1). Notably, both kidneys appeared normal without any suspicious lesions. The retroperitoneal mass caused a significant mass effect, compressing the right renal artery and the inferior vena cava. There were no other lesions suggesting metastases. Given the presence of a solitary retroperitoneal mass adjacent to the upper pole of the right kidney and adrenal gland, the initial radiological differential diagnosis included primary adrenal neoplasm, retroperitoneal sarcoma, lymphoma, and metastatic disease from an occult primary tumor. The absence of additional lesions on the staging CT scan made disseminated metastatic disease less likely at presentation, although a solitary metastasis from the kidney, lung, gastrointestinal tract, or melanoma could not be excluded. The location also raised the possibility of an exophytic renal tumor versus a primary lesion arising from perirenal fat or other retroperitoneal structures.

**Figure 2.**
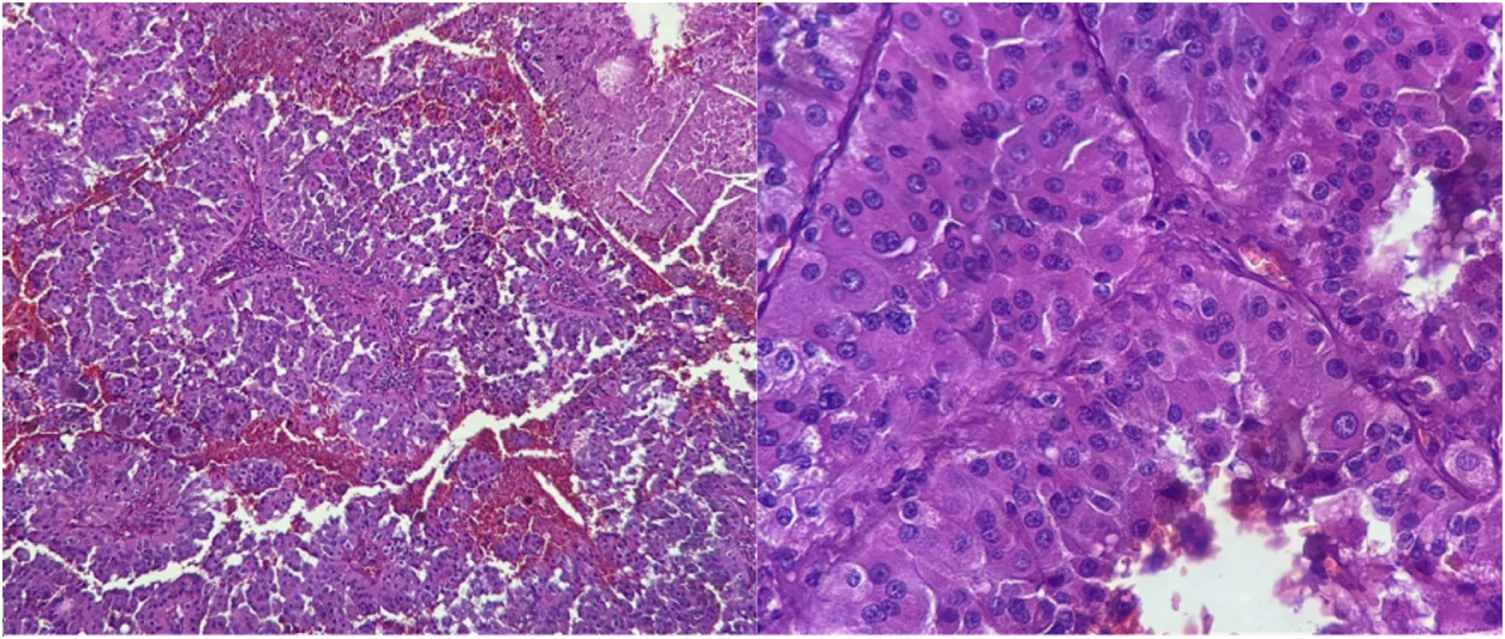
Microscopic view of the core biopsy. Malignant neoplastic epithelial proliferation is observed, with abundant papillary structures, large nuclei with prominent nucleoli, and moderate mitotic activity. The stroma is hemorrhagic and necrotic, with acute and chronic inflammation. H&E 100× (left) and H&E 200× (right).

On the day following admission, a CT-guided core biopsy of the retroperitoneal mass was performed; histopathology revealed a papillary renal cell carcinoma (Fig. 2). Immunohistochemistry of the biopsy was positive for PAX8, CAM 5.2, and AMACR, with focal positivity for PAX2, CK7, and pan-cytokeratin; it was negative for EMA, CK20, HMB45, TFE3, INSM1, GATA 3, and S100. The remaining diagnostic question was whether this represented an exophytic RCC, metastasis from an occult or regressed renal primary, or an extrarenal RCC without a renal primary. Serial cross-sectional imaging did not reveal any renal mass or additional lesions. The patient was referred for consultation to the surgical oncology department.

Following a multidisciplinary tumor board discussion, it was decided to resect the mass for diagnostic and treatment purposes. Laparoscopic resection of the retroperitoneal mass was performed without complications. The inferior vena cava and right adrenal vein were identified; the adrenal vein was clipped and divided for exposure. The tumor was found intraoperatively to be completely separable from both the kidney and adrenal gland. The retroperitoneal mass was dissected free from the right adrenal gland, the right kidney, perirenal fat, and renal vessels, then removed in an endobag. The operating time was 1 hour and 21 minutes. The postoperative course was favorable, and the patient was discharged on postoperative day 3. The pathologic report of the resected tumor was consistent with the core biopsy pathology report, confirming a type 2 papillary renal cell carcinoma with positive resection margins (pT1b R1).

**Figure 3.**
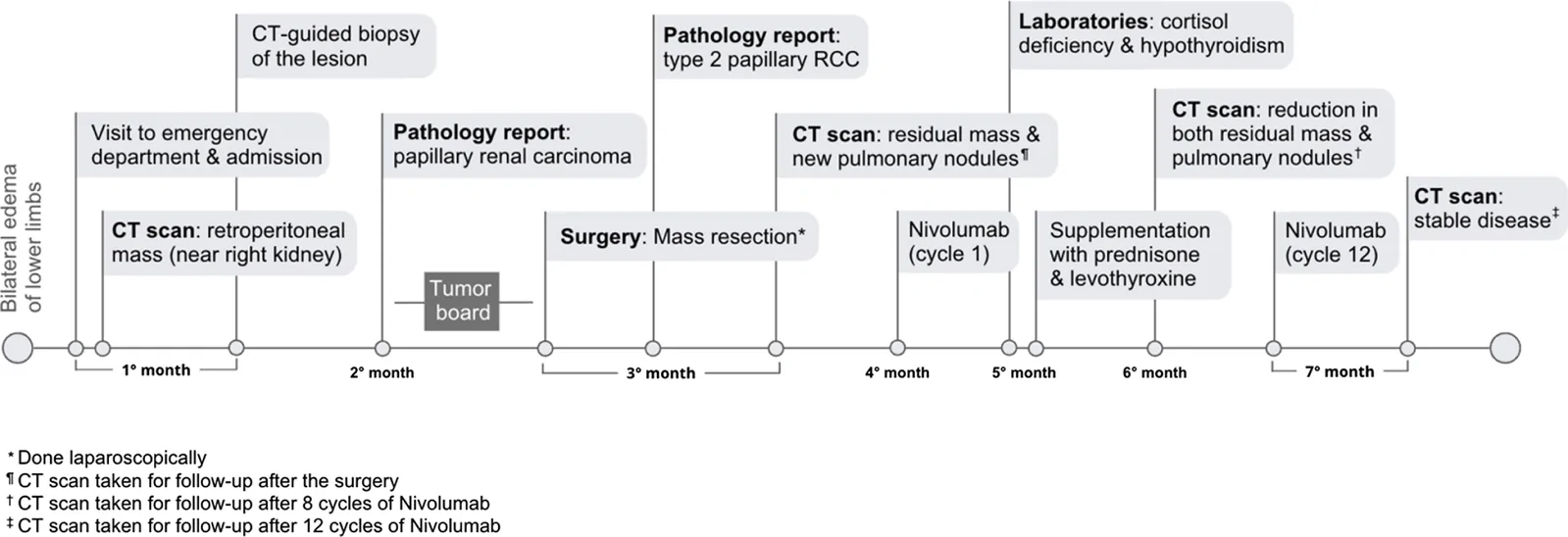
Timeline of key clinical events.

One month after surgery, CT imaging revealed multiple new bilateral pulmonary nodules (3–8 mm) and a 20 × 15 mm residual retroperitoneal lesion, consistent with disease progression. Nivolumab was initiated 2 months postoperatively with adequate tolerance. After four cycles, the abdominal lesion decreased to 14 mm, though the lung nodules showed radiologic progression; after eight cycles, a follow-up CT scan at 6 months demonstrated a reduction of pulmonary nodules and stability of the residual mass, indicating a partial response. During treatment, the patient developed immunotherapy-related cortisol deficiency and hypothyroidism, requiring prednisone and levothyroxine. He has now completed twelve nivolumab cycles, with imaging showing stable disease, and remains under close oncologic and endocrine follow-up (Fig. 3).

## Discussion

We describe a case of type 2 extrarenal pRCC, presenting as a solitary retroperitoneal mass without an identifiable renal primary.

The pRCC is the second most common RCC subtype, accounting for 15–20% of cases[1]. However, presentation without an identifiable renal primary is extremely rare[3]. Only nine cases of extrarenal pRCC without a renal primary have been reported, with four classified as type 2 (Table 1). In the absence of standardized treatment protocols, reported cases have involved heterogeneous management.

**Table 1:** Cases reported of extrarenal pRCC and extrarenal unclassified RCC without a renal primary.

Author	Year^a^	Sex	Age	Location	Pathologic subtype	Surgery	Systemic treatment	Outcomes
Sorscher *et al*[7]	2012	M	53	Neck, retroperitoneal & retrocrural lymphadenopathy + R adrenal gland	pRCC	**×**	✓Everolimus	No evidence of disease progression 10 months after systemic treatment was initiated.
Thamcha-roen *et al*[8]	2013	M	37	L supraclavicular lymph node + R lung apex + L neck + L common carotid & subclavian arteries + L thyroid lobe + L paraaortic nodes	pRCC	**×**	✓Sunitinib (once definite diagnosis was made)	Stable disease after 15 cycles of sunitinib.
							*Before definitive diagnosis: the disease progressed with 2 cycles of carboplatin + paclitaxel, and 2 cycles of cisplatin + etoposide*	
Costantino *et al*[9]	2016	M	68	Both adrenal glands + Liver (segment 8) + Bone lesion (T12)	Unclassified high-grade extrarenal RCC	✓Resection of the masses^b^	✓Sunitinib + Palliative radiotherapy (T12)	The disease remained clinically stable at 1 year after starting sunitinib.
				*Note: VHL mutation*				
Nagasaka *et al*[10]	2016	M	41	Case 1: Bilateral supraclavicular lymph nodes + R paratracheal lymph node + lymph nodes in subcarinal region + L lung + Bilateral cervical lymph nodes + Bone (diffuse)	pRCC	✓Excisional biopsy of lymph nodes (×3)	✓Pazopanib (RT before definitive diagnosis)	Partial response after 6 weeks.
		M	79	Case 2: Upper abdominal, retroperitoneal & bilateral pelvic lymph nodes + lower thoracic & upper lumbar vertebrae	pRCC	✓Excisional biopsy of lymph nodes (x3)	✓Sunitinib	Mild improvement of bilateral lower extremity swelling. Disease progression was not assessed.
							Before definitive diagnosis: AUC + paclitaxel	
Heary *et al*[11]	2019	M	54	Epidural tumor (at level of T2)	Unclassified extrarenal RCC	✓Tumor resection (3 surgical interventions)	**×**	Presence of diffuse metastatic disease. The patient lived 6 months following the third surgery.
				*Note: additional chromosome 7 & loss of chromosome Y*				
Nunes *et al*[12]	2019	M	68	Both adrenal glands (metachronous involvement)	pRCC	✓Bilateral adrenalectomy (metachronic approach)	**×**	Six months after surgery, the patient remained in oncologic remission.
Li *et al*[13]	2019	M	63	Retroperitoneal mass (lateral to inferior vena cava)	Type 2 pRCC	✓Mass resection	**×**	Five months after surgery, no recurrence was detected by imaging.
Srivishnu *et al*[14]	2021	M	34	L supraclavicular lymphadenopathy	Type 2 pRCC	**×**	✓Tyrosine kinase inhibitor (palliative)	The patient was unable to continue management; response could not be assessed.
							*Note: Non-affordability for immunotherapy*	
Mansoor *et al*[3]	2022	M	77	Case 2: Both adrenal glands	Unclassified extrarenal RCC	✓Right adrenalectomy	**×**	Twenty months after surgery, no evidence of local recurrence or metastatic disease detected by imaging.
								The patient died from glioblastoma multiforme 32 months after adrenalectomy.
Enes *et al*[15]	2024	M	41	Para-aortic & supraclavicular lymphadenopathy	Type 2 pRCC	✓Supraclavicular lymph node excision	✓Sunitinib	Metastatic lesions regressed after treatment was initiated.
								The patient died due to COVD-19 during systemic treatment.
Sarafi *et al*[16]	2024	F	57	Pelvic mass	Type 2 pRCC	✓En bloc resection of the mass^b^	**×**	Six months after surgery, no evidence of recurrence.

ccRCC, clear-cell renal cell carcinoma; F, female; L, Left; M, male; pRCC, papillary renal cell carcinoma; R: Right.

^a^ Year of publication.

^b^ Done by laparotomy; if not specified, the surgery was done laparoscopically.

Differentiating a primary extrarenal pRCC from metastatic disease relies on imaging, pathology, and clinical features[3]. Pathology confirms pRCC lineage (e.g., diffuse AMACR positivity and focal CK7) but cannot distinguish primary from metastatic origin[2]. Findings such as a solitary extrarenal mass, no additional metastases, and lack of renal parenchymal involvement support a primary tumor[3].

Most reported cases were managed with open surgery or simple excisional biopsies (Table 1). Our case demonstrates the feasibility of laparoscopic resection despite compression of major vascular structures (inferior vena cava and renal artery), with a short operative time and minimal morbidity. This minimally invasive approach may be considered in selected cases, which could allow faster recovery and earlier initiation of systemic therapy.

Our case differs from previous cases by presenting a solitary retroperitoneal mass adjacent to the right adrenal gland, later developing pulmonary metastases. Li *et al* described a similar retroperitoneal mass lateral to the inferior vena cava, treated with surgical resection without adjuvant therapy and without recurrence at 6 months[13].

As no guidelines address extrarenal pRCC without a renal primary, immunotherapy was selected by extrapolating histology-based NCCN recommendations[4]. The CheckMate 374 trial demonstrated an objective response rate of 22.7% (95% CI: 14.8–32.3) in patients with non-clear cell RCC[5]. The response observed in our patient aligns with emerging evidence supporting immune checkpoint inhibitors in non-clear cell RCC.

The development of immune-related endocrinopathies, including hypothyroidism and cortisol deficiency, is consistent with the known toxicity profile of immune checkpoint blockade[17]. Early recognition and hormone replacement allowed the continuation of immunotherapy without dose reduction, highlighting the importance of multidisciplinary management.

In our case, surgical resection followed by immunotherapy upon disease recurrence resulted in disease stabilization at a 6-month follow-up. The aggressive behavior observed – rapid development of pulmonary metastases and local recurrence within 1 month after resection – suggests behavior similar to that reported in type 2 pRCC[18].

## Conclusion

Extrarenal papillary renal cell carcinoma without a renal primary is extremely rare and difficult to diagnose. This case highlights the role of combined clinical, imaging, and pathological assessment. Although evidence regarding optimal treatment is limited, available reports suggest that surgical resection followed by immunotherapy may achieve better outcomes compared with immunotherapy alone.

## Data Availability

The data are not publicly available due to patient privacy but can be obtained from the corresponding author upon reasonable request.

## Patient perspective

I knew something was wrong when I developed swelling in my legs and had trouble walking. Overall, I have felt well and remain able to perform my daily activities. My recovery has been gradual, and immunotherapy side effects were detected through blood tests rather than symptoms.
